# Crystal structure of (1*Z*,4*Z*)-2,4-dimethyl-3*H*-benzo[*b*][1,4]diazepine

**DOI:** 10.1107/S2056989017004972

**Published:** 2017-04-04

**Authors:** Carla I. Nieto, Rosa M. Claramunt, M. Carmen Torralba, M. Rosario Torres, Jose Elguero

**Affiliations:** a Departamento de Química Orgánica y Bio-orgánica, Facultad de Ciencias, Universidad Nacional de Educación a Distancia (UNED), Senda del Rey 9, E-28040 Madrid, Spain; b Departamento de Química Inorgánica I, Facultad de Ciencias Químicas, Universidad Complutense de Madrid, E-28040 Madrid, Spain; c CAI Difraccion de Rayos X, Facultad de Ciencias Químicas, Universidad Complutense de Madrid, E-28040 Madrid, Spain; d Instituto de Química Medica, Centro Química Orgánica Manuel Lora-Tamayo,(CSIC), Juan de la Cierva, 3, E-28006 Madrid, Spain

**Keywords:** crystal structure, benzo[*b*][1,4]diazepine, 1,5-benzodiazepine

## Abstract

The title compound, C_11_H_12_N_2_, is not planar due to the folding of the seven-membered ring. In the crystal, mol­ecules are packed opposite each other to minimize the electronic repulsion but the long inter­molecular distances indicate that no directional contacts are found.

## Abstract 2

The title compound is not planar due to the folding of the seven-membered ring. In the crystal, mol­ecules are packed opposite each other to minimize the electronic repulsion but the long inter­molecular distances indicate that no directional contacts are found.

## Chemical context   

(1*Z*,4*Z*)-2,4-Dimethyl-3*H*-benzo[*b*][1,4]diazepine, C_11_H_12_N_2_ (Me, Me) (**1**), also called a 1,5-benzodiazepine, is a mol­ecule situated at the crossroad of many avenues of chemistry. This compound is associated with the names of Douglas Lloyd and Donald R. Marshall of the University of St Andrews in Scotland (Gibson *et al.*, 2002 ▸). These authors reported the synthesis of **1**, determined that its tautomeric structure is **1** and not **1′**, and also determined that the protonation of **1** yields the cation **1H^+^** (Lloyd *et al.*, 2002 ▸). For mol­ecules such as **1H^+^** they introduced the term ‘quasi-aromatic’ (Lloyd & Marshall, 1971 ▸), a term that has not survived the authors (Claramunt *et al.*, 2013 ▸). The inversion barrier of the seven-membered ring of **1** was measured to be 48.9 kJ mol^−1^ (Mannschreck *et al.*, 1967 ▸); our calculated value is 43.4 kJ mol^−1^ (Clara­munt *et al.*, 2013 ▸).
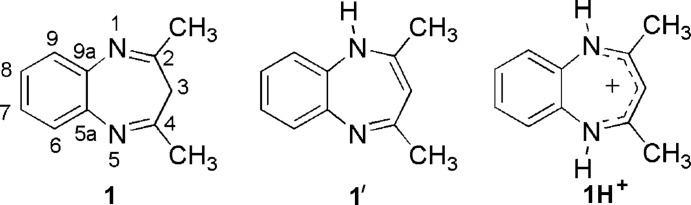


Benzo[*b*][1,4]diazepines continue to be the subject of many studies, but with other substituents (Bonacorso *et al.*, 1996 ▸; El-Azab, 2013 ▸; Aastha *et al.*, 2013 ▸; Solan *et al.*, 2014 ▸; Young *et al.*, 2016 ▸). Of the different procedures existing in the literature to prepare compound **1**, we used the reaction of acetyl­acetone with *o*-phenyl­enedi­amine using silica-supported sulfuric acid as catalyst under solvent-free conditions (Chen *et al.*, 2009 ▸). In spite of what the authors described in the paper, the reaction was not complete at room temperature and it was necessary to heat up to 273 K to attain a qu­anti­tative yield of the product, which was purified by column chromatography on silica gel and crystallized from ethyl acetate/hexane solution presenting a melting point of 408 K. We report herein on its characterization by ^1^H, ^13^C and ^15^N NMR in solution and solid state spectroscopy and, since its X-ray mol­ecular structure is unknown, we decided to complete the panorama of compound **1** determining it. Note that the structures of the monomethyl compound (Me, H) and the parent compound (H, H) are unknown, as well as those of their salts.

## Structural commentary   

**Figure 1:**
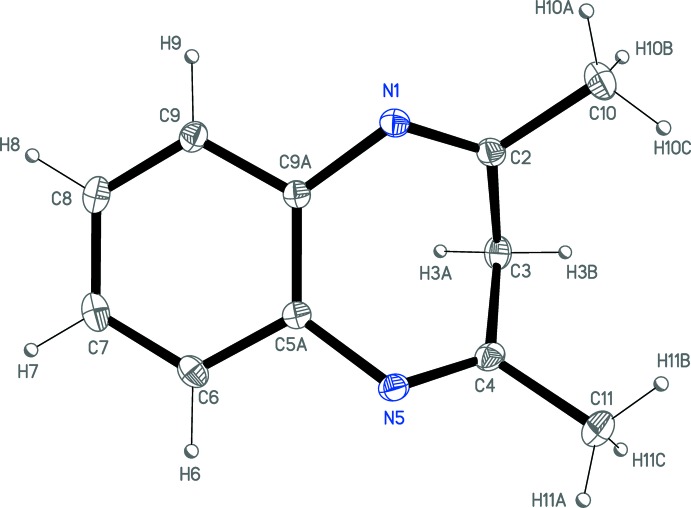
*ORTEP* plot (20% probability displacement ellipsoids) of **1.**

The title compound **1** crystallizes in the monoclinic space group *P*2_1_/*c* with one mol­ecule in the asymmetric unit (Fig. 1 ▸). As expected, the derivative is not planar due to the folding of the seven-membered ring. According to the electronic distribution for the two imine groups N1—C2 and N5—C4 [bond distances = 1.283 (3) and 1.281 (3) Å, respectively], atoms C2, C3 and C4 together with the two methyl groups of the diazepine ring deviate from the phenyl ring plane: atom C3 shows the largest displacement at 1.495 (1) Å while C2 and C4 are situated symmetrically at about 0.58 Å from it. The dihedral angle between the phenyl ring and C2/C3/C4 fragment of the diazepine ring is 87.8 (2)°, giving rise to a boat conformation for the diazepine ring.

**Figure 2:**
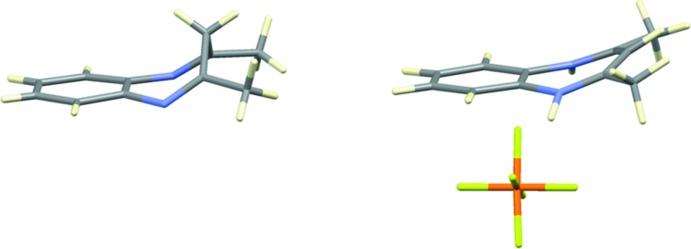
Comparative views of the seven-membered rings in **1** (left) and **1H^+^** (right) in the salt C_11_H_13_N_2_^+^·PF_6_^−^ (Blake *et al.*, 1991 ▸).

It is noteworthy that this is the only example found in the CSD database (Groom *et al.*, 2016 ▸) of a neutral diazepine derivative. For this reason, this structure is compared with the reported cationic diazepines C_11_H_13_N_2_^+^·*X* ^−^ [*X* = PF_6_^−^ (Blake *et al.*, 1991 ▸), Cl^−^ (Speakman *et al.*, 1976 ▸; Svensson & Timby, 1981 ▸) and ZnI_4_^2−^ (Orioli & Lip, 1974 ▸)] showing relevant structural differences. In the latter compounds, there is electronic delocalization in the N1/C2/C3/C4/N5 moiety that results in an almost planar geometry of this part of the seven-membered ring. However, in the neutral species, the C3 atom keeps both hydrogen atoms in an *sp* ^3^ conformation, leading to localization of the double bonds between the nitro­gen atoms and their adjacent carbon atoms, which induces a great deviation of this moiety from planarity (Fig. 2 ▸).

## Supra­molecular features   

**Figure 3:**
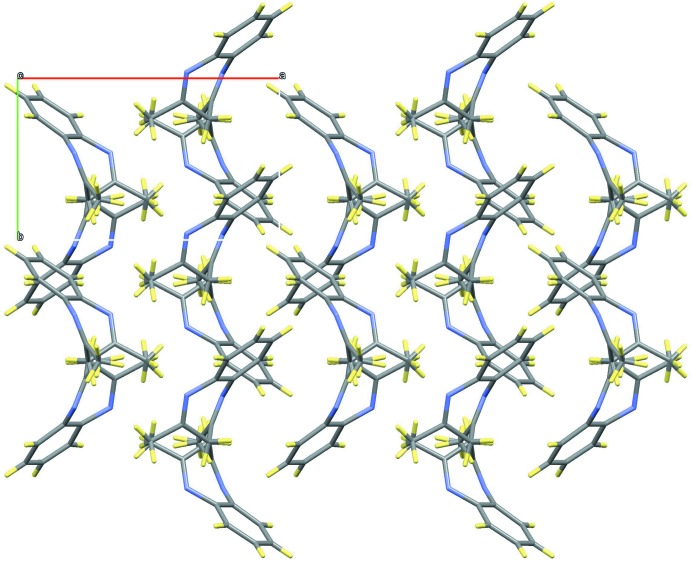
View of the crystal packing of **1**.

In the crystal, the mol­ecules are packed opposite each other to minimize electronic repulsion but the long inter­molecular distances indicate that no relevant contacts are found (Fig. 3 ▸). This feature differs from the salts previously mentioned, where the presence of the hydrogen atoms on the nitro­gen atoms allows the formation of N—H⋯*X* hydrogen bonds, leading to different supra­molecular networks. The absence of these atoms in **1**, along with the boat conformation described above, prevents the formation of any supra­molecular structure.

## Synthesis and crystallization   

All chemicals cited in the synthetic procedures are commercial compounds. Melting points were determined by DSC and thermograms (sample size 0.002–0.004 g) were recorded with a scan rate of 5.0 K min^−1^. Column chromatography was performed on silica gel 70–230 mesh. The NMR solution spectra were recorded on a 9.4 Tesla spectrometer (400.13 MHz for ^1^H, 100.62 MHz for ^13^C and 40.54 MHz for ^15^N) at 300 K with a 5 mm inverse detection H—*X* probe equipped with a *z*-gradient coil. Chemical shifts (δ in p.p.m.) are given from inter­nal solvents: CDCl_3_ 7.26 for ^1^H and 77.0 for ^13^C. Nitro­methane was used for ^15^N as external reference. CPMAS NMR spectra were obtained on a 9.4 Tesla spectrometer at 300 K (100.73 MHz for ^13^C and 40.60 MHz for ^15^N) using a 4 mm DVT probehead at spinning rates of 12 and 6 kHz, respectively. ^13^C spectra were originally referenced to a glycine sample and then the chemical shifts were recalculated to the Me_4_Si (for the glycine carbonyl atom δ = 176.1 p.p.m.) and ^15^N spectra to ^15^NH_4_Cl and then converted to the nitro­methane scale using the relationship: δ ^15^N (nitro­methane) = δ ^15^N (ammonium chloride) − 338.1 p.p.m.. Samples were spun at the magic angle at rates of 25 kHz and the experiments were carried out at 300 K.

**Synthesis of (1** ***Z*** **,4** ***Z*** **)-2,4-dimethyl-3** ***H*** **-benzo[** ***b*** **][1,4]diazepine (1):** To a mixture of 2,4-penta­nedione (100.12 mg, 1 mmol) and *o*-phenyl­enedi­amine (108.14 mg, 1 mmol), H_2_SO_4_·SiO_2_ (20 mg) was added. The mixture was heated with magnetic stirring at 373 K for 1 h. After completion of the reaction, the resulting black oil was purified using silica gel column chromatography (ethyl acetate/petroleum ether, 60:40) and crystallized from ethyl acetate/hexane solution to give colourless prisms (90%). *R*_f_ (ethyl acetate/petroleum ether 80:20): 0.25. M.p (DSC) 408 K (Nishio *et al.*, 1985 ▸, 403–405 K) ^1^H NMR (400.13 MHz, CDCl_3_) δ 7.36 (*dd*, ^3^ *J* = 6.0, ^4^ *J* = 3.5, 2H, H7, H8), 7.21 (*dd*, ^3^ *J* = 6.0, ^4^ *J* = 3.5 Hz, 2H, H6, H9), 2.82 (*br*, 2H, H3), 2.35 (*s*, 6H, CH_3_). ^13^C NMR (100.62 MHz, CDCl_3_) δ 157.6 (*q*, ^2^ *J* = 6.4 Hz, C2), 140.2 (*dd*, ^3^ *J* = 3 *J* = 7.0 Hz, C5a, C9a), 127.5 (*dddd* ^1^ *J* = 160.4, ^3^ *J* = 7.4, ^2^ *J* = ^2^ *J* = 3.5 Hz, C7, C8), 124.8 (*dd*, ^1^ *J* = 161.8, ^2^ *J* = 8.7 Hz, C6, C9), 43.2 (*tsep*,^1^ *J* = 134.5, 3 J = 2.8 Hz, C3), 27.6 (*qt*, ^1^ *J* = 128.0, ^3^ *J* = 2.5 Hz, CH_3_). ^15^N NMR (40.54 MHz, CDCl_3_) δ −74.1. ^13^C SSNMR (100.76 MHz, CPMAS) δ 162.9 and 161.5 (C2, C6), 142.2 (C5a, C9a), 128.0 (C7, C8), 125.5 and 124.6 (C6/C9), 43.1 (C3), 27.6 (CH_3_). ^15^N SSNMR (40.60 MHz, CPMAS) δ −69.7.

## Refinement   

Crystal data, data collection and structure refinement details are summarized in Table 1 ▸. Hydrogen atoms were included in their calculated positions (C—H = 0.93–0.97Å) and refined riding on the respective carbon atoms with *U*_iso_(H) = 1.2*U*_eq_(C) or 1.5*U*_eq_(C) for methyl H atoms.

## Supplementary Material

Crystal structure: contains datablock(s) global, I. DOI: 10.1107/S2056989017004972/hb7662sup1.cif

Structure factors: contains datablock(s) I. DOI: 10.1107/S2056989017004972/hb7662Isup2.hkl

Supporting information file. DOI: 10.1107/S2056989017004972/hb7662Isup3.mol

Supporting information file. DOI: 10.1107/S2056989017004972/hb7662Isup4.cml

CCDC reference: 1530551

Additional supporting information:  crystallographic information; 3D view; checkCIF report

## Figures and Tables

**Table 1:** Experimental details

Crystal data	
Chemical formula	C_11_H_12_N_2_
*M*_r_	172.23
Crystal system, space group	Monoclinic, *P*2_1_/*c*
Temperature (K)	296
*a*, *b*, *c* (Å)	11.8226 (16), 6.6305 (9), 13.3557 (19)
β (°)	114.531 (3)
*V* (Å^3^)	952.4 (2)
*Z*	4
Radiation type	Mo *K*α
μ (mm^−1^)	0.07
Crystal size (mm)	0.18 × 0.13 × 0.10

Data collection	
Diffractometer	Bruker SMART CCD
No. of measured, independent and observed [*I* > 2σ(*I*)] reflections	7529, 1876, 878
*R*_int_	0.072
(sin θ/λ)_max_ (Å^−1^)	0.617

Refinement	
*R*[*F* ^2^ > 2σ(*F* ^2^)], *wR*(*F* ^2^), *S*	0.051, 0.146, 0.99
No. of reflections	1876
No. of parameters	118
H-atom treatment	H-atom parameters constrained
Δρ_max_, Δρ_min_ (e Å^−3^)	0.18, −0.15

Computer programs: *SMART* and *SAINT* (Bruker, 2004 ▸), *SHELXS97*, *SHELXL97* and *SHELXTL* (Sheldrick, 2008 ▸).

## supplementary crystallographic information

### Crystal data

**Table:**

C_11_H_12_N_2_	*F*(000) = 368
*M**_r_* = 172.23	*D*_x_ = 1.201 Mg m^−^^3^
Monoclinic, *P*2_1_/*c*	Mo *K*α radiation, λ = 0.71073 Å
*a* = 11.8226 (16) Å	Cell parameters from 974 reflections
*b* = 6.6305 (9) Å	θ = 3.1–20.1°
*c* = 13.3557 (19) Å	µ = 0.07 mm^−^^1^
β = 114.531 (3)°	*T* = 296 K
*V* = 952.4 (2) Å^3^	Prismatic, colorless
*Z* = 4	0.18 × 0.13 × 0.10 mm

### Data collection

**Table:**

Bruker SMART CCD diffractometer	878 reflections with *I* > 2σ(*I*)
Radiation source: fine-focus sealed tube	*R*_int_ = 0.072
Graphite monochromator	θ_max_ = 26.0°, θ_min_ = 1.9°
phi and ω scans	*h* = −13→14
7529 measured reflections	*k* = −8→8
1876 independent reflections	*l* = −16→12

### Refinement

**Table:**

Refinement on *F*^2^	Primary atom site location: structure-invariant direct methods
Least-squares matrix: full	Secondary atom site location: difference Fourier map
*R*[*F*^2^ > 2σ(*F*^2^)] = 0.051	Hydrogen site location: inferred from neighbouring sites
*wR*(*F*^2^) = 0.146	H-atom parameters constrained
*S* = 0.99	*w* = 1/[σ^2^(*F*_o_^2^) + (0.060*P*)^2^] where *P* = (*F*_o_^2^ + 2*F*_c_^2^)/3
1876 reflections	(Δ/σ)_max_ < 0.001
118 parameters	Δρ_max_ = 0.18 e Å^−^^3^
0 restraints	Δρ_min_ = −0.15 e Å^−^^3^

### Special details

**Table:**

Geometry. All esds (except the esd in the dihedral angle between two l.s. planes) are estimated using the full covariance matrix. The cell esds are taken into account individually in the estimation of esds in distances, angles and torsion angles; correlations between esds in cell parameters are only used when they are defined by crystal symmetry. An approximate (isotropic) treatment of cell esds is used for estimating esds involving l.s. planes.
Refinement. Refinement of F^2^ against ALL reflections. The weighted R-factor wR and goodness of fit S are based on F^2^, conventional R-factors R are based on F, with F set to zero for negative F^2^. The threshold expression of F^2^ > 2sigma(F^2^) is used only for calculating R-factors(gt) etc. and is not relevant to the choice of reflections for refinement. R-factors based on F^2^ are statistically about twice as large as those based on F, and R- factors based on ALL data will be even larger.

### Fractional atomic coordinates and isotropic or equivalent isotropic displacement parameters (Å^2^)

**Table:**

	*x*	*y*	*z*	*U*_iso_*/*U*_eq_	
N1	0.3543 (2)	0.4619 (3)	0.32401 (16)	0.0491 (6)	
C2	0.3699 (2)	0.6414 (4)	0.2963 (2)	0.0490 (7)	
C3	0.2695 (3)	0.7496 (4)	0.2027 (2)	0.0551 (8)	
H3A	0.1911	0.7421	0.2095	0.066*	
H3B	0.2911	0.8904	0.2011	0.066*	
C4	0.2606 (2)	0.6429 (4)	0.1002 (2)	0.0511 (7)	
C5A	0.1784 (2)	0.3600 (4)	0.1489 (2)	0.0415 (6)	
N5	0.21672 (19)	0.4640 (3)	0.07648 (17)	0.0498 (6)	
C6	0.0737 (2)	0.2360 (4)	0.0995 (2)	0.0542 (7)	
H6	0.0330	0.2313	0.0231	0.065*	
C7	0.0299 (3)	0.1214 (4)	0.1614 (3)	0.0631 (8)	
H7	−0.0415	0.0439	0.1274	0.076*	
C8	0.0930 (3)	0.1223 (4)	0.2750 (3)	0.0602 (8)	
H8	0.0635	0.0462	0.3176	0.072*	
C9	0.1986 (3)	0.2348 (4)	0.3245 (2)	0.0502 (7)	
H9	0.2428	0.2284	0.4005	0.060*	
C9A	0.2416 (2)	0.3593 (3)	0.2635 (2)	0.0402 (6)	
C10	0.4921 (3)	0.7461 (5)	0.3555 (3)	0.0776 (10)	
H10A	0.5475	0.6599	0.4125	0.116*	
H10B	0.4795	0.8690	0.3875	0.116*	
H10C	0.5277	0.7765	0.3043	0.116*	
C11	0.3111 (3)	0.7471 (5)	0.0277 (2)	0.0799 (10)	
H11A	0.3008	0.6616	−0.0335	0.120*	
H11B	0.3979	0.7752	0.0692	0.120*	
H11C	0.2671	0.8713	0.0011	0.120*	

### Atomic displacement parameters (Å^2^)

**Table:**

	*U*^11^	*U*^22^	*U*^33^	*U*^12^	*U*^13^	*U*^23^
N1	0.0538 (15)	0.0523 (13)	0.0416 (13)	−0.0049 (12)	0.0203 (11)	−0.0059 (11)
C2	0.0515 (18)	0.0539 (17)	0.0451 (16)	−0.0062 (16)	0.0236 (14)	−0.0099 (15)
C3	0.0600 (19)	0.0378 (14)	0.073 (2)	0.0002 (14)	0.0327 (16)	0.0001 (15)
C4	0.0464 (17)	0.0558 (17)	0.0468 (17)	0.0072 (15)	0.0151 (13)	0.0147 (15)
C5A	0.0424 (16)	0.0413 (14)	0.0422 (16)	0.0054 (13)	0.0191 (13)	−0.0004 (13)
N5	0.0508 (14)	0.0570 (14)	0.0399 (13)	0.0007 (12)	0.0173 (11)	0.0043 (12)
C6	0.0423 (18)	0.0525 (16)	0.0603 (19)	0.0003 (14)	0.0138 (15)	−0.0073 (15)
C7	0.0497 (19)	0.0468 (17)	0.090 (3)	−0.0045 (14)	0.0267 (19)	−0.0044 (17)
C8	0.069 (2)	0.0457 (17)	0.080 (2)	−0.0021 (16)	0.0448 (19)	0.0037 (16)
C9	0.062 (2)	0.0426 (15)	0.0522 (17)	0.0007 (14)	0.0304 (15)	0.0014 (13)
C9A	0.0424 (15)	0.0377 (13)	0.0438 (16)	0.0018 (12)	0.0211 (13)	−0.0043 (13)
C10	0.064 (2)	0.080 (2)	0.081 (2)	−0.0249 (17)	0.0227 (18)	−0.0128 (18)
C11	0.089 (2)	0.087 (2)	0.069 (2)	−0.0131 (19)	0.0372 (19)	0.0183 (18)

### Geometric parameters (Å, º)

**Table:**

N1—C2	1.283 (3)	C6—H6	0.9300
N1—C9A	1.412 (3)	C7—C8	1.385 (4)
C2—C10	1.499 (4)	C7—H7	0.9300
C2—C3	1.502 (3)	C8—C9	1.367 (4)
C3—C4	1.505 (3)	C8—H8	0.9300
C3—H3A	0.9700	C9—C9A	1.396 (3)
C3—H3B	0.9700	C9—H9	0.9300
C4—N5	1.281 (3)	C10—H10A	0.9600
C4—C11	1.500 (4)	C10—H10B	0.9600
C5A—C9A	1.396 (3)	C10—H10C	0.9600
C5A—N5	1.407 (3)	C11—H11A	0.9600
C5A—C6	1.402 (3)	C11—H11B	0.9600
C6—C7	1.373 (4)	C11—H11C	0.9600
			
C2—N1—C9A	119.7 (2)	C6—C7—H7	120.3
N1—C2—C10	120.0 (3)	C9—C8—C7	120.1 (3)
N1—C2—C3	121.7 (2)	C9—C8—H8	120.0
C10—C2—C3	118.3 (3)	C7—C8—H8	120.0
C4—C3—C2	105.4 (2)	C8—C9—C9A	121.4 (3)
C4—C3—H3A	110.7	C8—C9—H9	119.3
C2—C3—H3A	110.7	C9A—C9—H9	119.3
C4—C3—H3B	110.7	C5A—C9A—C9	118.9 (2)
C2—C3—H3B	110.7	C5A—C9A—N1	125.0 (2)
H3A—C3—H3B	108.8	C9—C9A—N1	115.8 (2)
N5—C4—C3	121.9 (2)	C2—C10—H10A	109.5
N5—C4—C11	119.7 (3)	C2—C10—H10B	109.5
C3—C4—C11	118.3 (3)	H10A—C10—H10B	109.5
C9A—C5A—N5	125.2 (2)	C2—C10—H10C	109.5
C9A—C5A—C6	118.7 (2)	H10A—C10—H10C	109.5
N5—C5A—C6	115.9 (2)	H10B—C10—H10C	109.5
C4—N5—C5A	119.8 (2)	C4—C11—H11A	109.5
C7—C6—C5A	121.4 (3)	C4—C11—H11B	109.5
C7—C6—H6	119.3	H11A—C11—H11B	109.5
C5A—C6—H6	119.3	C4—C11—H11C	109.5
C8—C7—C6	119.4 (3)	H11A—C11—H11C	109.5
C8—C7—H7	120.3	H11B—C11—H11C	109.5
			
C9A—N1—C2—C10	−176.0 (2)	C5A—C6—C7—C8	−2.4 (4)
C9A—N1—C2—C3	1.9 (3)	C6—C7—C8—C9	−0.7 (4)
N1—C2—C3—C4	−70.2 (3)	C7—C8—C9—C9A	3.6 (4)
C10—C2—C3—C4	107.8 (3)	N5—C5A—C9A—C9	−174.3 (2)
C2—C3—C4—N5	70.2 (3)	C6—C5A—C9A—C9	0.3 (3)
C2—C3—C4—C11	−106.6 (3)	N5—C5A—C9A—N1	−0.5 (4)
C3—C4—N5—C5A	−1.8 (4)	C6—C5A—C9A—N1	174.1 (2)
C11—C4—N5—C5A	175.0 (2)	C8—C9—C9A—C5A	−3.3 (4)
C9A—C5A—N5—C4	−41.4 (4)	C8—C9—C9A—N1	−177.7 (2)
C6—C5A—N5—C4	143.8 (2)	C2—N1—C9A—C5A	42.0 (3)
C9A—C5A—C6—C7	2.6 (4)	C2—N1—C9A—C9	−144.0 (2)
N5—C5A—C6—C7	177.7 (2)		
